# Effects of Power Ultrasound on Stability of Cyanidin-3-glucoside Obtained from Blueberry

**DOI:** 10.3390/molecules21111564

**Published:** 2016-11-18

**Authors:** Guang-Long Yao, Xing-Hui Ma, Xian-Yin Cao, Jian Chen

**Affiliations:** College of Food Science and Technology, Hainan University, Haikou 570228, China; yaoguanglong@126.com (G.-L.Y.); MAH234@163.com (X.-H.M.); cxying_02@163.com (X.-Y.C.)

**Keywords:** ultrasonic waves, cyanidin-3-glucoside, stability, hydroxyl radicals, storage solvent

## Abstract

Power ultrasound (US) could potentially be used in the food industry in the future. However, the extent of anthocyanin degradation by US requires investigation. Cyanidin-3-glucoside (Cy-3-glu) obtained from blueberry extracts was used as research material to investigate the effect of power ultrasound on food processing of anthocyanin-rich raw materials. The effects of ultrasonic waves on the stability of Cy-3-glu and on the corresponding changes in UV-Vis spectrum and antioxidant activity were investigated, and the mechanisms of anthocyanin degradation induced by ultrasonic waves were discussed. To explore Cy-3-glu degradation in different environments, we kept the Cy-3-glu solution treated with ultrasonic waves in four concentrations (0%, 10%, 20%, and 50%) of ethanol aqueous solutions to simulate water, beer, wine, and liquor storage environment according to the chemical kinetics method. Results show that the basic spectral characteristics of Cy-3-glu did not significantly change after power ultrasound cell crusher application at 30 °C. However, with anthocyanin degradation, the intensity of the peak for Cy-3-glu at 504 nm significantly decreased (*p* < 0.05). The degradation kinetics of Cy-3-glu by ultrasonic waves (200–500 W frequency) fitted well to first-order reaction kinetics, and the degradation rate constant of Cy-3-glu under power ultrasound was considerably larger than that under thermal degradation (*p* < 0.05). The sensitivity of the anthocyanins of blueberry to temperature increased with increasing ethanol concentration, and the longest half-life was observed in 20% ethanol aqueous solution.

## 1. Introduction

Ultrasonic cavitation during food processing instantaneously produce high temperatures and causes pressure changes in liquid solutions, which can kill some bacteria, inactivate viruses, and even disrupt small microbe cell walls, thereby extending the freshness and maintaining the original flavor of food [1,2]. Ultrasound-assisted extraction has many characteristics, such as shortened extraction time, no heating, which can avoid the thermal damage of effective components in the crude extract, it is suitable for extracting thermosensitive materials [3,4]. Meanwhile, the content of effective components in crude extracts is higher, it can provide favorable conditions for further refining, it is conforms to the principles of green extraction [5]. In addition, reducing the dosage of solvent can effectively lower the cost in the extraction process. Physiological activity of most effective components is basically unaffected, which can improve the quality of the product. Therefore, technology of ultrasonic power-assisted extraction is widely used in the food industry [6].

Research shows that the anthocyanin-retaining effect of appropriate power ultrasound in juice was remarkable. Chen reported that when raspberry anthocyanins were extracted with 400 W ultrasonic power in 200 s, the total anthocyanin content reached 78.13% [7]. Compared with the traditional method, it saved much time and solvent. Tiwari studied the effects of ultrasonic treatment on anthocyanins in grape juice. The retention rate of anthocyanins in grape juice reached the maximum at 20 kHz ultrasonic processing [8]. However, Stojanovic determined that high frequency ultrasound had an adverse impact on blueberry anthocyanins and phenolic substances [9]. Tiwari’s research showed that anthocyanins in strawberry juice would degrade when it is subjected to high frequency ultrasound; however, the highest degradation rate was 3.2% or less [10]. High power ultrasound propagates in liquids, which will then cause the bubble cavitation phenomenon due to the change in pressure. High ultrasonic power can produce not only cavitation but also chemical reactions, including free radicals and other reactions [11]. These reactions could result in the degradation of anthocyanins and color changes in some products, especially in berry products (such as strawberry juice, blueberry juice, and blueberry wine), which are rich in anthocyanins [12,13]. Therefore, gaining further understanding on the effect of power ultrasound on the stability of anthocyanins is necessary to protect anthocyanin-rich products and to induce the ultrasonic degradation mechanism of anthocyanins.

Cyanidin-3-glucoside (Cy-3-glu), which is the major anthocyanin compound in blueberry was used as research material to avoid the interference of other ingredients and of temperature [14,15]; in addition, we performed our experiments strictly at a temperature of 30 °C by using ice to cool the solutions down. The influencing factors of ultrasonic degradation of Cy-3-glu according to degradation phenomena were analyzed under the condition of low temperature, spectral characteristic changes, degradation kinetics, concentration of free radicals, and antioxidant activity of anthocyanin degradation.

## 2. Results and Discussion

### 2.1. HPLC Measurement of Cy-3-glu in Blueberries

**Figure 1:**
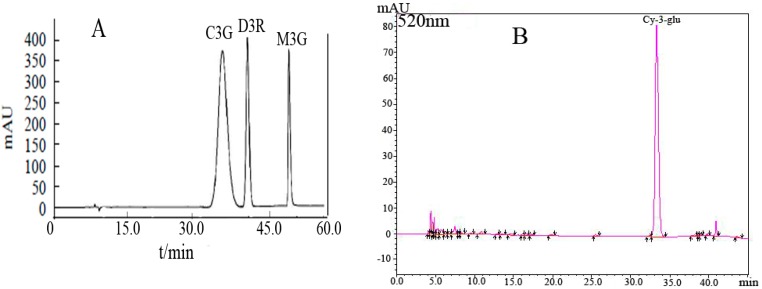
(**A**) HPLC chromatogram of anthocyanin standards; (**B**) HPLC chromatogram of Cy-3-glu in blueberries. Notes: delphinidin-3-rutinoside (D3R); cyanidin-3-glucoside (C3G); malvidin-3-galactoside (M3G).

The standard anthocyanin monomers (cyanidin-3-glucoside, delphinidin-3-rutinoside and malvidin-3-galactoside) were used to identify the unknown peaks in the Cy-3-glu in blueberries of blueberries (Figure 1). The concentration measurements of each sample were repeated three times, and the peak area (Y) and concentration (X) were analyzed using linear regression analysis.

### 2.2. Analysis of Spectral Characteristics of Cy-3-glu in Blueberries

**Figure 2:**
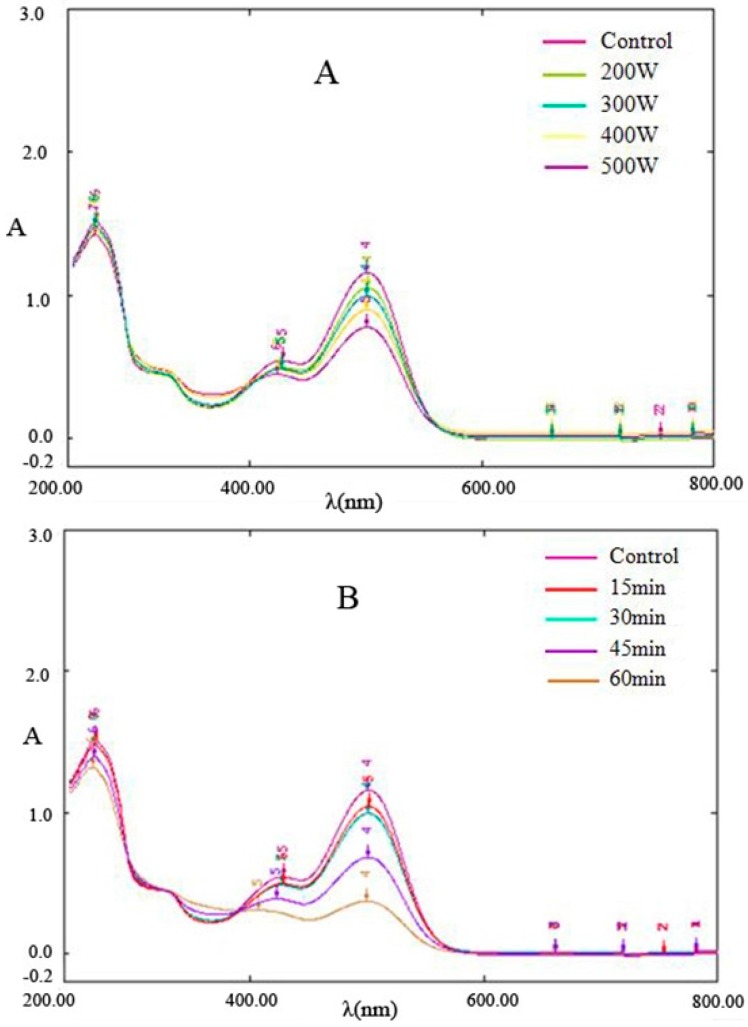
Effect of ultrasonic power and time on the spectral characteristics of Cy-3-glu (*n* = 3). Note: (**A**) 30 min processing; (**B**) 300 watt power.

Figure 2 shows the wavelength scanning spectrum diagram of Cy-3-glu after power ultrasound application. The main absorption peaks were observed at 285 and 504 nm, and a small peak near 340 nm and a shoulder peak near 430–440 nm were noted. The characteristics of UV-Vis spectrum of Cy-3-glu were similar to those reported by Fa’ tima [16] and Torgils [17]. Studies have shown that the 285 nm spike peak and 400–600 nm wide peak are the characteristic absorptions of cationic anthocyanins, 330–340 nm represents unsaturated carbonyl compounds in chalcone, and 440 nm spike peak is the characteristic absorption of a browning substance.

Figure 2 showed that the characteristics of the UV-Vis spectrum of Cy-3-glu after power ultrasound are similar to those of Cy-3-glu without treatment. However, with increasing ultrasonic power and prolonged treatment time, the absorption intensity of Cy-3-glu at 285 and 504 nm gradually declined, suggesting that power ultrasound degraded Cy-3-glu. Paula [18]studied the thermal degradation of cyanidin, delphinium, pelargonidin, and malvidin in acidic aqueous media and found that these anthocyanins generate chalcone during thermal degradation; the spectral characteristics at 285 and 504 nm dropped significantly after degradation, whereas the absorption at 325 nm was enhanced, in which two isobestic points were observed in the spectral curve. As shown in Figure 2, the spectral curve also contains two isobestic points, and the other characteristics were consistent with those of the abovementioned research. No obvious enhancement in absorption at 325 nm was observed, suggesting that the intermediate degradation did not involve chalcone; 295 nm absorption was obtained, and no obvious enhancement was observed, suggesting that hydroxybenzoic acid and hydroxybenzaldehyde were not generated. All of these results indicated that the pathway for Cy-3-glu degradation by power ultrasound is different from that under thermal treatment.

### 2.3. Dynamic Analysis of Cy-3-glu in Blueberries

Dynamic model analysis of anthocyanin degradation could be used to predict the rate of anthocyanin degradation to reduce loss of anthocyanins, which is useful in food quality control. Scholars use different models to simulate anthocyanin degradation induced by different factors; they found that anthocyanins in line with the degradation kinetics models were different under different conditions. They mainly could be divided into the zero, primary, secondary, and complex reaction kinetics. Tiwari [10] found that anthocyanin degradation in blueberry juice follows the zero-order reaction kinetics under ozone condition. Turfan [19,20] reported that anthocyanin degradation in blueberry juice, pomegranate juice, and cherry juice containing different concentrations of hydrogen peroxide fitted with first-order reaction kinetics, whereas the effect of different ascorbic acid concentrations on anthocyanin degradation in tart cherry juice is consistent with second-order reaction kinetics. Anthocyanins in blood orange juice were studied at 70–90 °C, and degradation of different concentrations of fructose, glucose, and sucrose could only be described by a complex reaction [21].

**Figure 3:**
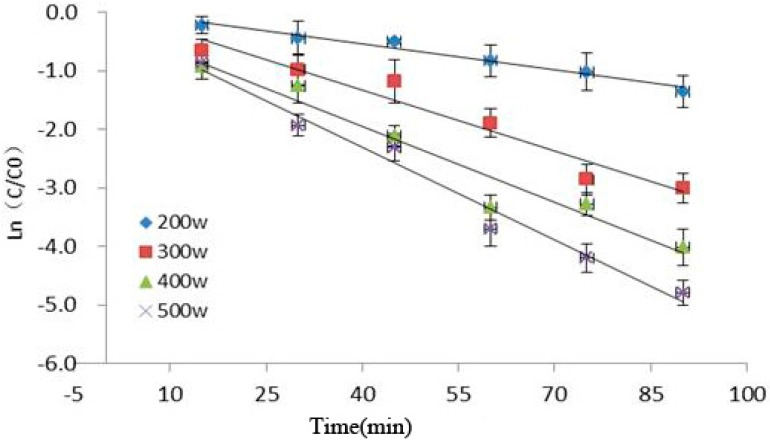
Kinetics analysis of Cy-3-glu after power ultrasound (*n* = 3).

Figure 3 showed the relationship among ultrasonic power, time, and Cy-3-glu degradation as observed in our experiment. Under increasing ultrasonic power and extended reaction time, the retention rate of Cy-3-glu significantly decreased, suggesting that Cy-3-glu was significantly degraded (*p* < 0.05). Cy-3-glu degradation induced by power ultrasound fitted with first-order reaction kinetics (*R*^2^ > 0.91), and the kinetics parameters are shown in Table 1.

The effect of power ultrasound on anthocyanins is mainly caused by cavitation, which produces high temperatures and pressures, converting water molecules into free radicals. These free radicals are highly reactive owing to their unpaired electrons, resulting in their strong oxidizing effect, further promoting anthocyanin degradation. Pyrolysis of water molecules requires much energy to generate free radicals [2,22]; ultrasonic pulse energy was low under low power condition, producing low amount of free radicals and thus increasing the retention rate of Cy-3-glu.

### 2.4. ·OH Determination

**Figure 4:**
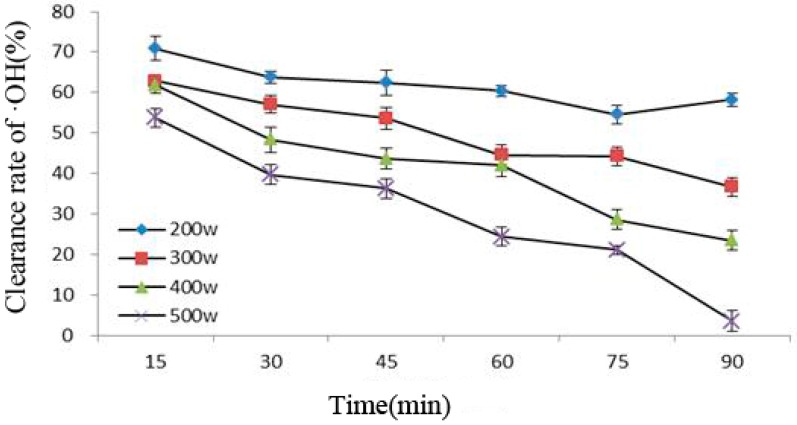
Correlation between clearance rate of ·OH and ultrasonic power and treatment time (*n* = 3).

Figure 4 shows that under the same ultrasonic power, the ability of Cy-3-glu to remove ·OH under increasing ultrasonic time gradually decreased; in addition, the greater the power, the stronger the influence would be. This phenomenon was caused by cavitation during power ultrasound, causing molecules to produce ·OH, which in turn attacked Cy-3-glu, resulting in the lost ability of the latter to scavenge free radicals. Thus, the ability of Cy-3-glu to remove ·OH decreased [23,24]; the greater the intensity of power ultrasound, the more severe the damage would be.

### 2.5. Analysis of Antioxidant Activity

**Figure 5:**
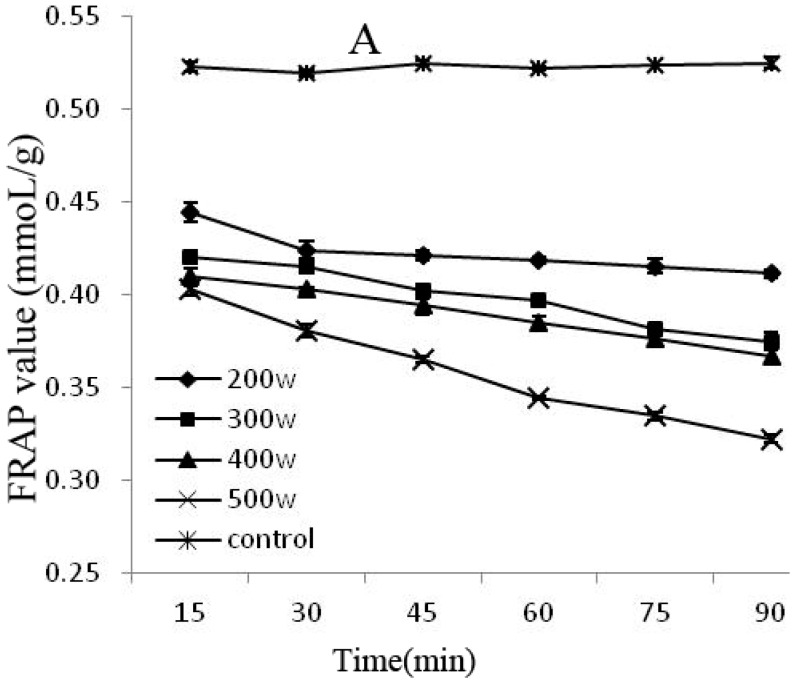

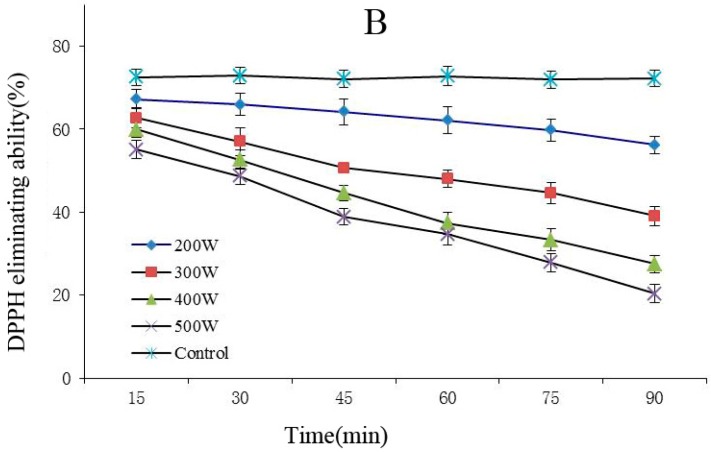
Effect of power ultrasound on the antioxidant activities of Cy-3-glu as measured by FRAP (**A**) and DPPH (**B**) (*n* = 3).

The FRAP and DPPH methods are rapid, simple, sensitive, and reproducible, whereas the beta carotene-linoleic acid method uses only a small amount of samples and reagents, making these methods advantageous [25]. Therefore, this experiment combined the FRAP, DPPH, and beta carotene-linoleic acid systems, which comprehensively evaluated the changes of the degradation of the anthocyanins antioxidant activity after power ultrasound. FRAP (A) and DPPH (B) activity changes in Cy-3-glu solution system after power ultrasound are shown in Figure 5. Compared with that in the control, the FRAP and DPPH antioxidant activity of Cy-3-glu solution system after power ultrasound decreased, and the trend was more significant under increased power and prolonged treatment (*p* < 0.05). 

**Figure 6:**
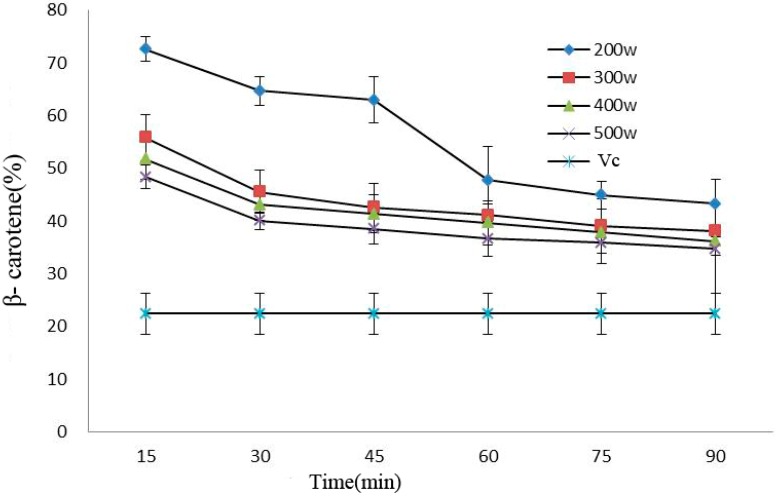
Effect of power ultrasound on the antioxidant activities of Cy-3-glu as measured by β-carotene (*n* = 3).

Processing power and time would affect the antioxidant activity of beta carotene in anthocyanins. Oxidation resistance declined under prolonged processing time, and such a decline became more significant under 200 W (*p* < 0.05). In addition, the antioxidant capacity of the anthocyanin solutions was considerably stronger than that of V_C_ solution after power ultrasound (*p* < 0.05) (Figure 6).

**Figure 7:**
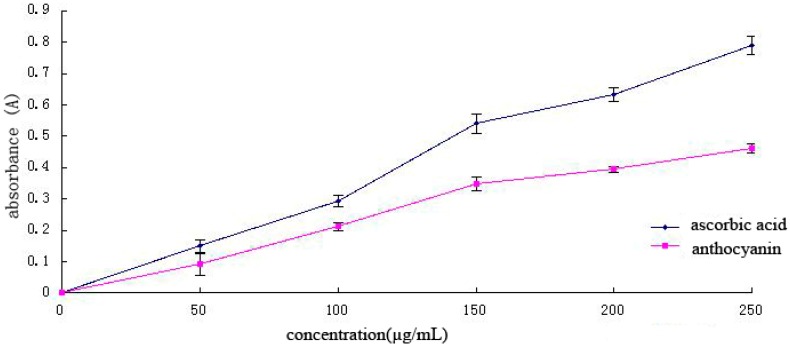
Total antioxidant capacity of different concentrations of ascorbic acid and Cy-3-glu solutions (*n* = 3).

The total antioxidant capacity is the sum of the effectiveness of different active ingredients to remove all kinds of free radicals; thus, total antioxidant capacity is an important indicator in evaluating the antioxidant function of bioactive substances [26,27]. Antioxidants in samples are positively correlated with decreasing power (Figure 7). With increasing Cy-3-glu solution concentration, the total antioxidant capacity also increased, although its capacity was weaker than that of ascorbic acid. The total antioxidant capacity of ascorbic acid was nearly two times than that of Cy-3-glu solution.

In our experiment, Cy-3-glu was significantly degraded after power ultrasound application. Antioxidant activity also declined compared with that under thermal degradation, indicating that the pathway for ultrasonic degradation and thermal degradation differs. Consistent with the result of spectral analysis, the residual anthocyanins after power ultrasound remained to be in the form of salt cationic (AH^+^, red). The degradation products were not hydroxybenzoic acid, hydroxyl-benzaldehyde, and chalcone, so the antioxidant activity did not [22,23]. Turfan [19] found that the main mechanism of anthocyanin degradation by H_2_O_2_ includes three reactions: ·OH generation and induction of the benzene of anthocyanins to generate CO_2_ and H_2_O [24]. Similar to previous findings, we found that ·OH concentration and Cy-3-glu degradation show a good correlation. Therefore, we concluded that the main mechanism of anthocyanin degradation induced by power ultrasound was cavitation, in which water molecules generate ·OH, which in turn attacked anthocyanins, leading to anthocyanin degradation:

H_2_O_2_→H^+^ + HOO^−^ (1)

HOOH→·OOH + ·H
(2)

HOOH→2·OH
(3)

### 2.6. Degradation Anthocyanins of Blueberry in Ethanol Solutions

Ethanol concentrations were set as 0%, 10%, 20%, and 50% to simulate the blueberry anthocyanins in water, beer, red wine, and white wine in a storage environment. Degradation dynamics of the residues of Cy-3-glu solution was analyzed at 4 (refrigeration), 25 (room temperature), 75 (pasteurization), and 95 °C (boiling water sterilization). The results showed that Cy-3-glu degradation followed first-order reaction kinetics.

**Figure 8:**
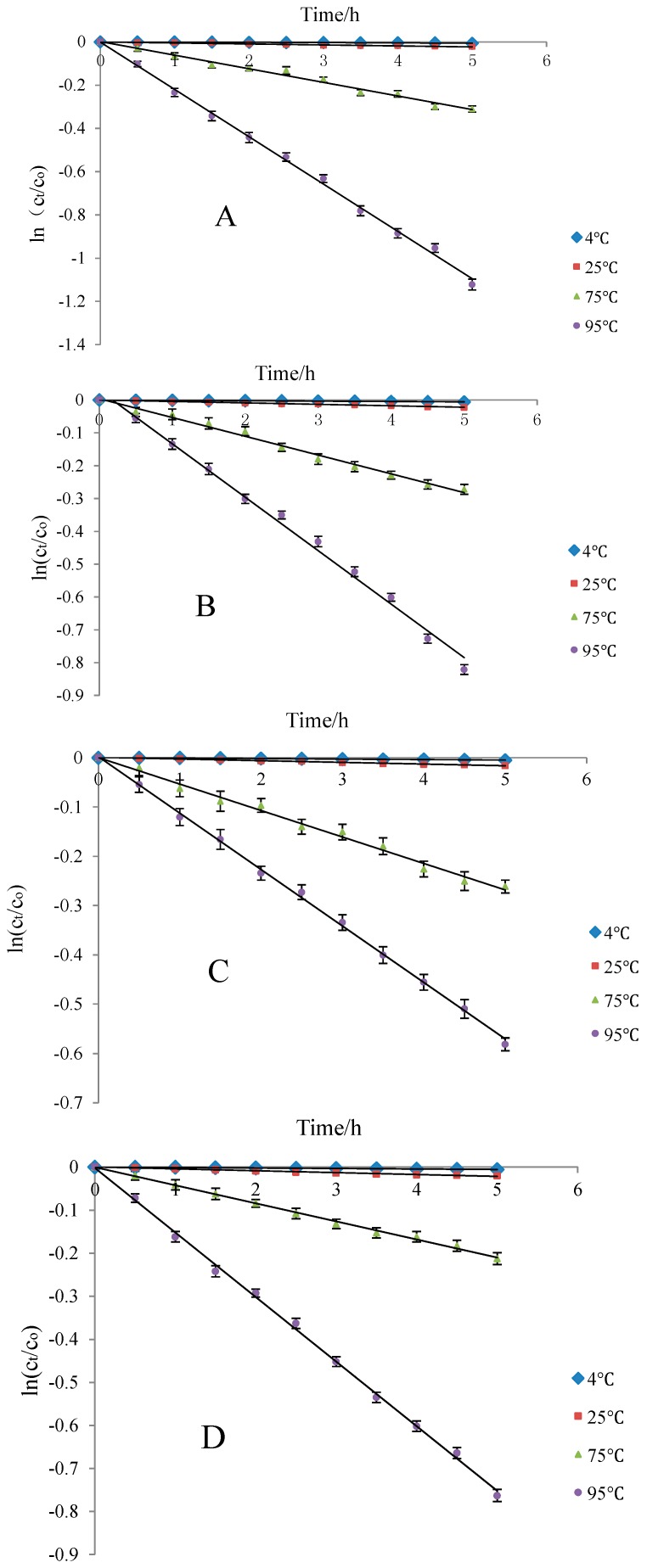
Thermal degradation of Cy-3-glu solution at different heating temperatures in different ethanol concentrations (*n* = 3). Note: deionized water (**A**); 10% ethanol (**B**); 20% ethanol (**C**); and 50% ethanol (**D**).

With increasing temperature, the trendline fitting coefficient also increased and gradually approached 1. The Cy-3-glu solution was stable under low temperature and short supply heating. Among the four kinds of storage solvents, t_1/2_ was longest under normal temperature and 20% ethanol solution (wine) storage environment, suggesting that blueberry anthocyanins can be better preserved under this condition (Figure 8).

## 3. Experimental Section

### 3.1. Materials and Reagents

Blueberries (bluecrop variety) were provided by Dandong Organic Food Co., Ltd. (Shenyang, Liaoning, China). Analytical-grade methanol, trifluoroacetic acid, bromocresol purple, ethanol, FeSO_4_, sodium phosphate, ammonium molybdate, sulfuric acid, anhydrous methanol, ascorbic acid, H_2_O_2_, HCl, beta carotene, chloroform, linoleic acid, Tween-20, 1,1-diphenyl-2-picrylhydrazyl radical (DPPH), acetate buffer, 2,4,6-tri-2-pyridinyl-1,3,5-triazine (TPTZ) and ethyl acetate were purchased from Beijing Chemical Reagent Co. (Beijing, China). Anthocyanin standard monomer, namely, cyanidin-3-glucoside (Chromadex 00011605, purity 98.5%) delphinidin-3-rutinoside (Chromadex 00016371, purity 98%) and malvidin-3-galactoside (Chromadex 00017345, purity 99%), were obtained from Polyphenol AS (Sandnes, Norway). and chromatographic grade formic acid and methanol, were obtained from Sigma-Aldrich Co. (Billerica, MA, USA). Amberlite XAD-7 resin column and Sephadex LH-20 gel resin were purchased from Polyphenol AS. Deionized water was used throughout the experiment.

### 3.2. Separation and Purification of Cy-3-glu

Blueberries (100 g) were crushed in a pounding machine; extraction was performed using 2 L of methanol containing 0.5% trifluoroacetic acid (TFA) for 24 h at 0 °C following centrifugation; rotary evaporation was performed to remove methanol at 38 °C, and concentrated anthocyanins were obtained. The concentrate containing ethyl acetate at 1:1 ratio (*v*:*v*) was subjected under liquid–liquid extraction for three times to remove fat soluble component. Amberlite–XAD 7 column chromatography was used for preliminary separation to remove impurities, such as sugar and the acid contained in pigment mixtures. Methanol containing 0.5% TFA was used to eluent concentration before rotary evaporation, and Sephadex LH-20 column chromatography was used for further purification. The chromatographic conditions were as follows: 5 mL of sample; methanol and water (7:3, *v*: *v*) containing 0.5% TFA were used as eluent; purification was performed below 4 °C; and eluent velocity was 0.3 mL/min. Four ribbons separated from the column and were collected in a brown bottle. The purity of the collected components was determined by HPLC, and component 3 (with 33.32 min peak time) was identified as Cy-3-glu by comparing with standard monomers (with 32.7–33.9 min peak time) [25,26].

### 3.3. Determination of Cy-3-glu Content of Cy-3-glu in Blueberries

Cy-3-glu solutions (10, 20, 30, 40, and 50 µmol/L) were mixed with formic acid, methanol, and water (3:47:50, *v*:*v*:*v*). Cy-3-glu content was detected with HPLC-DAD after 0.2 μm organic membrane filtration and darkening after 1 h. A drawing standard curve was then obtained. Sample determination was performed as follows: 1 mL of sample was analyzed on a Waters-e2695-HPLC-DAD system from Waters Co. (Milford, MA, USA) Agilent 1100 workstation from Aglilent Co. (Denver, CO, USA) equipped with 0.2μm organic membrane filter. The chromatographic conditions were as follows: The analyses were performed on a Diamonsil C_18_ column (250 mm × 4.6 mm, 5 μm), the flow rate was 1.0 mL/min, the column temperature was set at 35 °C, 20 µL of sample was analyzed at 520 nm; mobile phase A contains 5% formic acid aqueous solution, and mobile phase B consists of formic acid, methanol, and water (5:45:50, *v*:*v*:*v*). The elution program was as follows: 40% B gradient elution for 3 min at 0.4 mL/min flow rate; then B phase was increased from 40% to 60% within 24 min at 0.4 mL/min flow rate; B phase gradient was subsequently increased from 60% to 100% within 5 min at 0.8 mL/min velocity; finally, B phase gradient was reduced to its original concentration of 40%; baseline was balanced for 8 min [27].

### 3.4. Power Ultrasound of Cy-3-glu in Blueberries

Cy-3-glu in blueberries (5 mL) was placed in a Scientz-IID power ultrasound cell crusher from Ningbo biotechnology Co. (Ningbo, Zhejiang, China); the entire process was performed strictly at 30 °C to eliminate the effect of temperature on anthocyanin degradation. Four power gradients (200–500 W) and six time gradients (15–90 min) were set [28,29]; samples without power ultrasound were prepared for comparison in terms of dynamics, spectral characteristics, antioxidant activity, and hydroxyl radical (·OH) concentration.

### 3.5. Spectral Feature Analysis of Cy-3-glu in Blueberries

Solution of Cy-3-glu in blueberries was spectrally scanned three times before and after power ultrasound at room temperature. The scanning wavelength ranged from 200 nm to 800 nm, and the scan interval was 1 nm [30,31].

### 3.6. Dynamic Analysis of Cy-3-glu in Blueberries

First-order reaction kinetics model was used to analyze ultrasonic degradation of Cy-3-glu in blueberries [32,33]:

Ln (C_t_/C_0_) = −k × t (4)

t_1/2_ = −Ln 0.5 × k^−1^ (5)

where C_t_: retention rate for Cy-3-glu in blueberries after power ultrasound at time t; C_0_: Cy-3-glu in blueberries content of untreated sample. k: rate constant for the first-order reaction kinetics; t_1/2_: half time of degradation of Cy-3-glu in blueberries.

### 3.7. In Vitro Antioxidant Activity of Cy-3-glu in Blueberries

#### 3.7.1. Removal of ·OH by Cy-3-glu in Blueberries

Bromocresol purple (50.0 mg) was accurately weighed and diluted in 95% ethanol solution up to 100 mL; FeSO_4_ (273.6 mg, 1.8 mmol/mL) was dissolved in distilled water in a 100 mL volumetric flask; 30% H_2_O_2_ (6 mL) was accurately measured and diluted with heavy steam water up to 100 mL. Subsequently, 0.05% bromocresol purple solution (0.4 mL), 0.1 mol/L HCl (0.5 mL), and 1.8 mmol/mL FeSO_4_ (0.5 mL) were placed in separate test tubes, diluted with heavy steam water under vigorous shaking, and then reacted with water for 8 min at 30 °C. The absorbance of the solution was determined three times at 420 nm and then averaged, denoted as A_0_. Afterward, 0.5 mL of 0.1 mol/L H_2_O_2_ was added into 1 mmol/L FeSO_4_ solution, and absorbance was determined three times; the average was denoted as A. Low absorbance value was calculated as follows [29,31]:

△A = A_0_ − A (6)

#### 3.7.2. Removal of DPPH by Cy-3-glu in Blueberries after Power Ultrasound

About 2 mL of 2 × 10^−4^ mol/LDPPH solution (prepared with 95% ethanol) was added to 2 mL Cy-3-glu solution after ultrasound application with varying power frequencies. The solutions were mixed and reacted away from the light at room temperature. The absorbance values of the mixed solution at 517 nm were determined for three times in parallel, with 2 mL ethanol solution + 2 mL DPPH solution as the control group:

DPPH clearance rate = [(A_0_ − A_1_)/A_0_] × 100% (7)

where A_0_ was the absorbance of control group and A_1_ was the absorbance of Cy-3-glu group.

#### 3.7.3. Determination of Ferric Reducing Antioxidant Potential Assay of Cy-3-glu in Blueberries after Power Ultrasound

At pH 3.6, 300 mmol/L acetate buffer (3.1 g trihydrate sodium acetate and 16 mL acetic acid soluble were dissolved in 1 L distilled water) and 10 mmol/L TPTZ solution (prepared with 40 mmol/L HCl and 20 mmol/L FeCl_3_ solution) were blended according to the volume ratio of 10:1:1 and insulated at 37 °C for 30 min to obtain the ferric reducing antioxidant potential assay (FRAP) solution. Afterward, 0.3 mL Cy-3-glu solution was added into 5.7 mL FRAP solution after ultrasound at varying frequencies. The solution was then placed in 30 min in the dark after blending. The absorbance value at 593 nm wavelength was determined for three times in parallel. The absorbance (A) was denoted as the *x*-axis, whereas different molarities of FeSO_4_ (mmol/L) were used as the *y*-axis to draw the FeSO_4_ standard curve, i.e., *y* = 0.2858 + 0.0294*x*, *R*^2^ = 0.9989. The FRAP values of the Cy-3-glu solution were according to the FeSO_4_ standard curve with the same absorbance.

#### 3.7.4. Role of Cy-3-glu in Blueberries in Beta Carotene–Linoleic Acid System

Beta carotene (5 mg) was accurately weighed, dissolved in chloroform (10 mL), and then mixed with linoleic acid (0.25 mL) and Tween-20 (2.0 mL). The mixture was transferred into a round bottom flask and subjected under rotary evaporation at 50 °C for 4.0 min. Next, 500 mL of distilled water was added. The mixture served as reaction solution. In addition, 0.5 mg/mL V_C_ solution was used for comparison, and Cy-3-glu in blueberries was used as standard solution. The standard solution (1.0 mL) and reaction solution (4.0 mL) were mixed in a test tube and allowed to react in a water bath at 50 °C. Absorbance was measured at 470 nm every 25 min for a total of 150 min (distilled water instead of beta carotene was used as blank). Antioxidant capacity was calculated by the following formula [26,31]:

Antioxidant capacity (%) = [1 − (A_0_ − A_t_)/(A_0_’ − A_t_’)] × 100% (8)

where A_0_ and A_t_ are the absorbance of Cy-3-glu in blueberries at 0 and 150 min, respectively; and A_0_’ and A_t_’ are the absorbance in the absence of Cy-3-glu in blueberries at 0 and 150 min, respectively.

#### 3.7.5. Determination of Total Antioxidant Capacity in Cy-3-glu in Blueberries

Ammonium molybdate was used to determine antioxidant capacity. Sodium phosphate (2.13 g), ammonium molybdate (0.99 g), and sulfuric acid (0.99 mL) were dissolved in water (200 mL), mixed, and diluted with anhydrous methanol; the concentration gradients of Cy-3-glu in blueberries ranged from 50 μg/mL to 250 μg/mL. Diluted Cy-3-glu in blueberries (0.5 mL) was mixed into the ammonium molybdate (5 mL) reaction system and heated at 90 °C for 90 min; and absorbance of the reaction liquid at 695 nm was measured after the reaction system cooled to room temperature. The measured total antioxidant capacity of anthocyanins was reflected with corresponding Vc concentration [30,31].

### 3.8. Degradation Test of Cy-3-glu in Blueberries

Cy-3-glu in blueberries powder (0.1 g) was weighed and dissolved up to a constant volume of 50 mL (pH = 3) in deionized water and 10%, 20%, 50% ethanol at 4 , 25, 75 , and 95 °C, respectively. Samples (2 mL) were obtained from each group every 30 min and then immediately cooled in ice water. As mentioned in Section 2.3, the degradation dynamics must be analyzed to determine the variation in Cy-3-glu concentration [32,33].

### 3.9. Statistical Analysis

Experiments were performed in triplicate, final data were expressed as the mean ± standard deviation ($\bar{x}$ ± s). ANOVA was employed to assess the differences among groups. Statistical analysis was performed using SPSS 21.0 from IBM Co. (Armonk, NY, USA) .Significant differences were denoted by *p* < 0.05.

## 4. Conclusions

The basic spectral characteristics of Cy-3-glu solution did not change obviously after power ultrasound exposure, and the absorption at 501 nm decreased significantly with anthocyanin degradation. Degradation of Cy-3-glu by power ultrasound fitted first-order kinetics. The rate of anthocyanin degradation induced by power ultrasound was consistently greater than that observed under thermal degradation. Under prolonged power ultrasound and increased power, ·OH concentration increased, and the antioxidant activity of anthocyanins decreased. In addition, ·OH concentration showed a good correlation with Cy-3-glu degradation. This result indicated that cavitation is the main mechanism of ultrasonic degradation of anthocyanins; cavitation caused water molecules to produce ·OH, which attacked anthocyanins, resulting in open-loop degradation of anthocyanins. Degradation of anthocyanins of blueberry followed first-order reaction kinetics, and the anthocyanins of blueberry showed the longest storage time under 20% ethanol solution (wine) at room temperature.

## Figures and Tables

**Table 1:** Kinetics parameters of Cy-3-glu degradation exposed to ultrasonic wave.

Parameters	200 W	300 W	400 W	500 W
K (min^−1^)	1.34 × 10^−2^	3.59 × 10^−2^	5.06 × 10^−2^	5.89 × 10^−2^
t_1/2_ (min)	51.7274	19.3077	13.6986	11.7682
*R^2^*	0.9709	0.9475	0.9491	0.9782

## Abbreviations

Cy-3-glu: Cyanidin-3-glucoside
FRAP: Ferric reducing/antioxidant power
DPPH: 1,1-Diphenyl-2-picrylhydrazyl radical
